# Tumor aggression among hepatitis-C related hepatocellular carcinoma patients: an observational study regarding the impact of anti-HCV therapy

**DOI:** 10.1186/s13027-020-00300-z

**Published:** 2020-05-27

**Authors:** Javeria Khalid, Mohammad Umar, Tofeeq Ur-Rehman, Mashhood Ali, Gul Majid Khan

**Affiliations:** 1 Department of Pharmacy, Quaid-i-Azam University, Islamabad, 45320 Pakistan; 2 Clinical Pharmacist at Shifa International Hospital Islamabad, Islamabad, Pakistan; 3 Center for Liver and Digestive Diseases, Holy Family Hospital, Rawalpindi Medical University, Rawalpindiand, 46300 Pakistan; 4 Gasteroenterology Department, Pakistan Institute of Medical Sciences (PIMS) Hospital, Islamabad, 44000 Pakistan

**Keywords:** Hepatocellular carcinoma, HCV related HCC, Aggressiveness index, Tumor pattern

## Abstract

**Background:**

Hepatitis C virus (HCV) represents a major risk factor for hepatocellular carcinoma (HCC) development and anti-HCV therapy is a significant measure to reduce the incidence of HCC, however development of HCC in HCV treated patients is an emerging clinical problem which needs to be investigated. In this study we aim to analyze association between anti-HCV therapy and tumor pattern of HCV related HCC patients.

**Methods:**

Hepatocellular Carcinoma (HCC) patients with seropositivity for hepatitis C virus (HCV) antibodies, registered at three tertiary care hospitals of Rawalpindi and Islamabad, Pakistan during August 2017 to July 2018 were enrolled. Selected patients were then segregated in two groups on the basis of their HCV treatment history i.e., “TN” (HCV Treatment Naïve i.e. having no history/medical record for treatment prior to HCC diagnosis) and “TH” (Treated for HCV infection). Aggressiveness index (AgI) scoring system was applied to determine the tumor pattern. Univariate and multivariate analysis was carried out to analyze the independent effect of anti-HCV therapy on tumor pattern.

**Results:**

Out of 234 consecutive HCC patients, 171 HCV-related HCC patients were enrolled in final analysis and labeled as “TN” (*n* = 120) and “TH” (*n* = 51). Tumor pattern was found to be significantly aggressive (*P* = 0.02) in the treated cohort with an adjusted odds of 2.47 for aggressive and 6.92 for highly aggressive tumor. Neutrophil to lymphocyte ratio (NLR) was strongly associated with highly aggressive tumor pattern (*P* = 0.012). Patients in TN group were found to be marginally older than those in the TH group (59.5 vs. 55 years) where mean age of the patients treated with direct acting anti-viral agents was found to be visibly lower than mean age of patients who received interferon based treatment (53.5 vs. 57 years) with significant masculine predominance (62.1 vs. 37.9%, *P* = 0.049).

**Conclusion:**

We observed raised neutrophil to lymphocyte ratio and prominence of younger age with aggressive tumor biology in HCV treated HCC patients. These observations highlight the need for a longitudinal prospective study on HCV positive subjects treated with antivirals, irrespective of treatment response.

## Background

Hepatocellular carcinoma (HCC) accounts for 700,000 new cases worldwide with mortality rate of > 90% [1]. It is the fourth leading cause of cancer related deaths and ranked sixth in incident cases [2]. Lack of early screening and effective surveillance programs make poor the overall prognosis of HCC even in developed countries where survival for > 5 years is observed in only 12% of cases [3] and patients with aggressive HCC phenotype have significantly reduced rate of survival [4]. Tumor aggressiveness factors and liver function parameters have been shown to independently influence the survival of HCC patients and hence are incorporated in many classification systems such as Okuda, BCLC (Barcelona Clinic Liver Cancer) and CLIP (Cancer of the Liver Italian Program) [5–7]. Aggressiveness Index (AgI) is a recently introduced scoring system for HCC tumor patterns which takes accounts of four tumor-related parameters i.e., maximum tumor diameter (MTD), number of tumor nodules, portal vein thrombosis (PVT) and serum alpha-fetoprotein (AFP) level. Studies have shown that higher AgI score is associated with poor prognosis [8, 9].

Chronic viral hepatitis, autoimmune disease, consumption of aflatoxin contaminated commodities and excessive alcohol intake are some of the many etiological factors of HCC. The chronic viral hepatitis caused by hepatitis B & C viruses (HBV & HCV, respectively) is the most common risk factor that accounts for approximately 80% of HCC cases worldwide [10]. Globally the major share of HCC is followed in Asian and African countries where HBV and HCV infections are more prevalent [11]. Previously HBV related HCC patients were 3–4 times more than all of the other viral hepatitis related HCC cases, however HCV related HCC is on rise these days due to higher prevalence and poor control of HCV infection [12, 13]. Chronic hepatitis C (CHC) affects 170 million people worldwide and in 20–30% cases it progresses to cirrhosis [14, 15]. Pakistan is second to Egypt in the list of high prevalence countries of the world for CHC infection with 4.9% prevalence where majority of HCC cases (60–70%) are attributed to HCV infection [16–18]. There are several reports aimed to associate HCV genotype with evolution, prognosis and therapy response to chronic liver disease; however, no consensus has yet been established [19–21].

Unlike HBV-related HCC, the HCV-related HCC usually appears with cirrhotic liver morphology similar to HDV related HCC [22–24]. Although the exact mechanism of HCV infection in HCC development is unknown, it is considered that HCV involves both direct viral effect through NS53 core protein and indirect pathway through cytokines, steatosis induction and oxidative stress [19]. Though HCV circulate in body, it specifically infects hepatocytes and escapes the adaptive and innate immune system of the host. After incubation of 2–12 weeks, an acute phase appears that leads to viral clearance spontaneously. If HCV is not cleared, acute HCV infection turns into chronic HCV infection. This CHC infection leads to liver cirrhosis in response to long term inflammation resulting from the host immune response against HCV infection [25].Thus, the primary step in prevention of HCV-related HCC is to control the development of cirrhosis via anti-viral treatment and subsequent monitoring under regular surveillance programs. Previously, parenteral interferon (IFN) with ribavirin was the standard therapy for CHC [26] with success rate of 40–50% in the treated cases [27]. In 2013, direct acting antiviral agents (DAAs) were approved for oral anti-HCV therapy with improved treatment outcomes i.e., sustained virological response (SVR) rate in > 90% cases [28]. Although the chance to develop HCC greatly reduced when CHC was treated at early stage [29] however, the risk was not eliminated completely [30, 31]. Biannual follow-up for RNA level and liver morphology is strongly recommended to identify the HCV-infection relapse and detection of HCC at an early stage [32, 33]. The prognosis of early stage HCC is far better than late stage HCC where the health care team is left with no option but the palliative or the supportive care [34, 35].

In 2016, a debate was initiated regarding the impact of anti-HCV therapy on occurrence and recurrence of HCC after the publication of a report by Conti et al. [36], however in recently published reviews scientists concluded that DAAs do not appear to increase the risk for HCC occurrence while the recurrence rate needs to be elaborated further in larger cohorts [37, 38]. Initial studies have analyzed the occurrence and recurrence of HCC after achieving SVR; however the impact of anti-HCV therapy on tumor pattern didn’t get any attention. Therefore, in this study we aim to investigate the association of anti-HCV therapy with the onset of symptomatic HCC and tumor patterns in terms of AgI among HCV related HCC patients in Pakistan.

## Methodology

### Patients’ enrollment and data collection

This comparative-exploratory study is based on the data of patients diagnosed with HCC as per AASLD (American Association for the Study of Liver Diseases) criteria [32], between August 2017 to July 2018, at the liver centers and gastroenterology departments of three tertiary care hospitals located in the twin cities, Rawalpindi and Islamabad, Pakistan. HCC patients with serum positivity for HCV-antibody were included in the study, while the details of HCV anti-body testing are specified in the supplementary Table 2. Patients having history of co-infection with other types of viruses (e.g. HBV), alcohol consumption and history of any other cancer were excluded. HCV related HCC patients who received anti HCV treatment but failed to respond the therapy were also excluded from the final analysis. On predesigned data sheet, the information regarding demographics, HCV diagnosis, type of anti HCV treatment, liver function parameters (ALT, ALKP, total bilirubin, serum albumin level), platelets count, WBC’s, neutrophil count, lymphocyte count, liver morphology (through ultra sound scans), cirrhosis and diabetes status was collected from patient’s current/previous medical records or through one-to-one interviews, wherever required. Cirrhosis was defined on basis of ultrasound-based cirrhosis scale developed by Hung et al. [39]. On the basis of patients’ current medical record, the Child-Turcotte-Pugh (CTP) class [40] and BCLC stage [7]were defined and noted. Neutrophil-to-lymphocyte ratio (NLR) was calculated and taken as an inflammatory index along with hypoalbumanemia. On the basis of medical record history of anti HCV treatment with IFN/DAA based therapy, the enrolled HCC patients were segregated in two groups viz. “TN” (HCV Treatment Naive, having no history/medical record for treatment prior to HCC diagnosis) “and TH” (Treated for HCV infection using interferon/DAA based regimens).

### Analysis of tumor aggression pattern

Tumor pattern was noted based on contrast enhanced triple phase CT scans for size, diameter and number of tumor nodules. Serum AFP level (ng/mL) was measured by electrochemiluminisence immunoassay using Autoanalyser Cobas e411 while considering < 7 ng/ml as reference value with coefficient of variation as ≤5%. Previously reported aggressiveness index (AgI) was applied to determine the tumor pattern on the basis of AFP level, number of tumor nodules, maximum tumor diameter and portal vein thrombosis (PVT) [29]. AFP level (ng/mL) were given the score of 1, 2 and 3 for AFP <  100, AFP 100–1000 and AFP >  1000, respectively. A score of 1 or 3 was assigned for number of tumor nodules ≤3 or >  3, respectively. Maximum tumor diameter (MTD) was assigned the score of 1, 2 and 3 for MTD <  4.5, 4.5–9.6 and >  9.6 cm, respectively. The presence and absence of PVT was given a score of 3 and 1, respectively (8, 9, 41). The overall sum was defined as AgI score which was divided into non-aggressive (AgI score = 4), aggressive (AgI score = 5–8) and highly-aggressive (AgI score > 8) tumor. The Ethics Committee and Institutional Review Boards of concerned hospitals/institution approved the study and study protocol conforms to the ethical guidelines of the 1975 Declaration of Helsinki. The verbal/written consent was obtained from the participating patients.

### Statistical analysis

Primary data was recorded in Microsoft Excel 2010 and secondary data was generated with anonymous patient coding. Data was analyzed using the SPSS® (IBM version 20). Continuous variables were reported as mean and standard deviation (SD) while categorical variables were expressed as frequencies and percentages (%). Baseline patient characteristics were compared between TN and TH groups and categorical variables were analyzed using Pearson’s chi-square test of association while testing the hypothesis for significant association *p*-value < 0.05 was considered significant. Univariate analysis was performed using logistic regression to analyze the difference in frequency of different parameters between TN and TH group. The results were presented as crude odds (cOR) with 95% confidence interval (CI). Multiple logistic regression model was further applied to analyze the independent effect of HCV-treatment on tumor pattern. In multivariate model all those variables were included having *p*-value ≤ 0.10 in univariate analysis. Receiver operating curve (ROC) analysis was performed to define the cut-off value for NLR. Kolmogorove-Smirnov test was performed to test for normality of data. Comparison of liver function parameters and laboratory values on basis of aggressiveness index categories was performed by using one-way analysis of variance (ANOVA) or non-parametric Kruskal-Wallis tests whichever suits best.

## Results

**Fig. 1:**
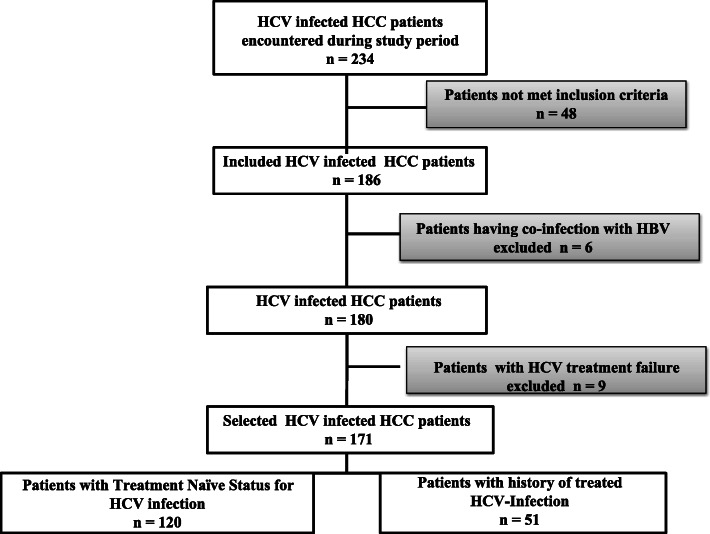
Description of HCC patients included in the study

Overall, 234 HCC patients who underwent visit in the selected hospitals during the study period, showed serum positivity for HCV antibodies. Among these, 186 cases met inclusion criteria. Six patients having co-infection with HBV and nine patients with failed HCV treatment were then excluded from the final analysis (Fig. 1).

### Baseline patient characteristics of HCV-related HCC patients

Base line characteristics of 171 HCV related HCC patients (TN = 120, TH = 51) included in the analysis are depicted in Table 1. Majority of the patients were diagnosed for HCC symptomatically (85.4%), where all were cirrhotic with a greater proportion of male gender (63.2%). Most of the patients in treated group had taken DAA based anti-HCV therapy (62.7%). For HCC patients in TH group, the overall mean duration between CHC treatment and HCC diagnosis was 34.55 ± 30.13 months (Table 1). The duration was smaller for DAA treated group as compared to IFN treated group (13.05 ± 7.35 vs. 70.74 ± 15.03 months).

**Table 1:** Baseline characteristics of enrolled patients

Parameter	Value
Sex (M) (%)	108 (63.2)
Age (yr) (mean ± SD)	57.72 ± 7.95
Tobacco Consumers (yes) (%)	98 (57.3)
Cirrhosis (yes) (%)	171 (100)
DM (yes) (%)	52 (30.4)
Mode of HCC Diagnosis	
Symptomatic (%)	146 (85.4)
Incidental (%)	16 (9.4)
Screening (%)	9 (5.2)
TH Patients (%)	51 (29.8)
INF-α based treatment (with or without Ribavirin) (%)	19 (37.3)
DAA based treatment (%)	32 (62.7)
Duration between HCV treatment and HCC diagnosis (months) (mean ± SD)	34.55 ± 30.13
Duration between treatment with IFN and HCC (months) (mean ± SD)	70.74 ± 15.03
Duration between treatment with DAA and HCC (months) (mean ± SD)	13.05 ± 7.35
AgI score of HCV Treatment Naïve Group (median, range)	6.0 (4–12)
AgI score of HCV Treatment Group (median, range)	8.0 (4–11)

*TH* Treated for HCV infection using interferon/DAA based regimens (*n* = 51); *DM* Diabetes Mellitus; *HCV* Hepatitis-C Virus; *INF-α* Interferon-alpha; *DAA* Direct Acting Anti-virals (including: sofosbuvir and/or daclatasvir); *AgI* Aggressiveness Index (sum of score) = Maximum Tumor Diameter (MTD) (in tertiles): MTD <  4.5; 4.5 = MTD = 9.6; MTD >  9.6; scores 1, 2, 3 respectively; Alpha-fetoprotein (AFP) (cut-off): AFP <  100; 100 = AFP = 1000; AFP >  1000; scores 1, 2, 3 respectively; Portal Vein Thrombosis (PVT) (no/yes): PVT (no); PVT (yes); scores 1, 3 respectively; nodules (number): Nodules = 3; nodules > 3; scores 1, 3 respectively

### Comparison of TN and TH groups

**Fig. 2:**
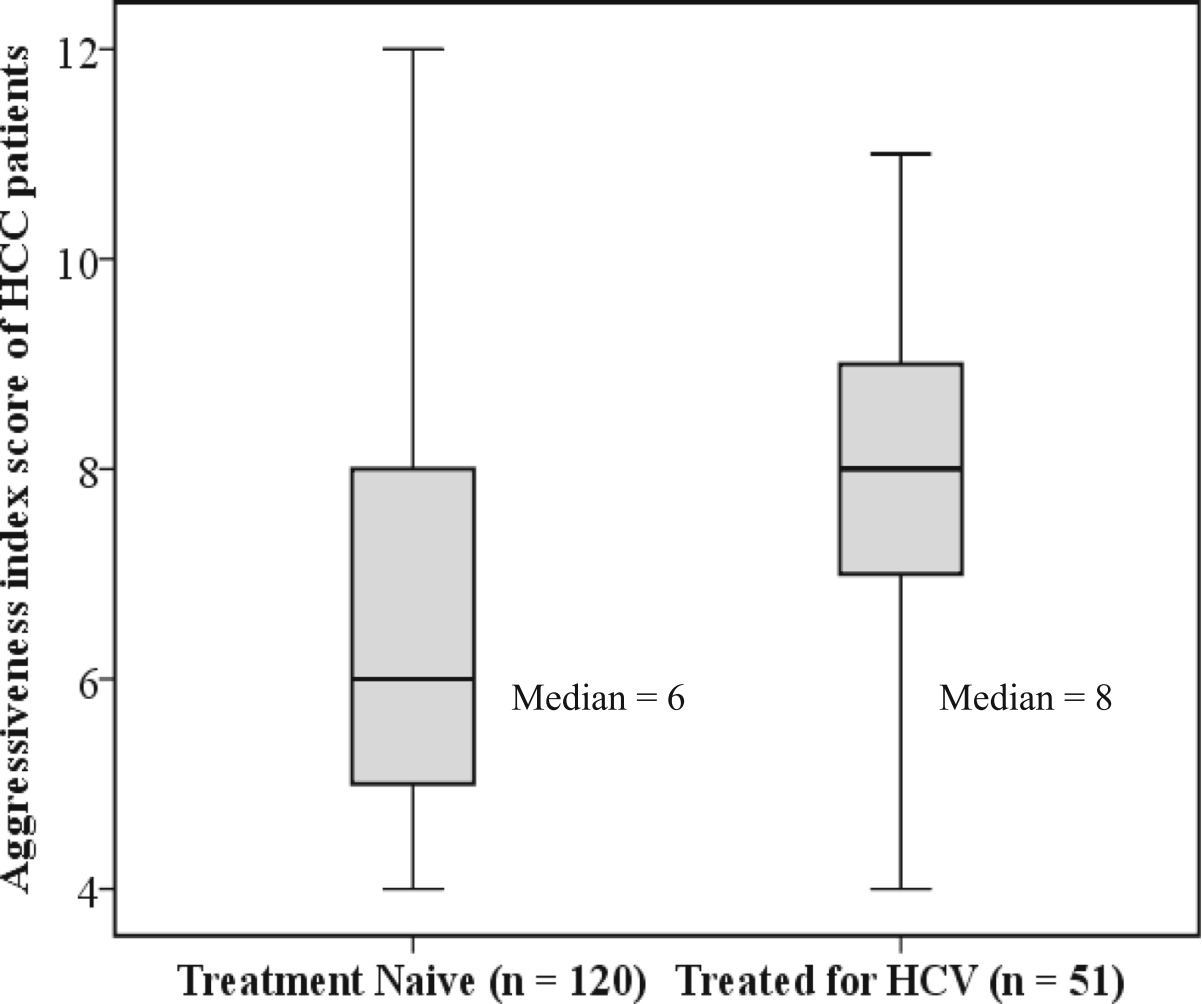
Aggressiveness index score distribution among HCV Treatment Naïve and HCV Treated patients

There was no apparent difference in gender distribution, tobacco consumption and DM status between the two groups (Table 2). Advanced cirrhosis was more prominent in patients having history of complete viral eradication with anti-viral therapy as compared to patients in TN group (Table 2). The risk of hypoalbumenemia was 1.22 times greater for TH group in reference with TN group (Table 3). Out of various laboratory parameters, the platelet count and NLR of two groups were found to be significantly different with 3.11 times greater risk for low platelet count (95% CI: 1.38–6.97, *P* = 0.005) and 2.26 times greater risk for raised NLR in TH group (95% CI: 1.05–4.84, *P* = 0.0335) (Table 2). Majority of the patients (51%) in TH group were presented with terminal cancer stage with double of estimated risk as compared to TN group (cOR: 2.06 CI: 0.76–5.59, *P* = 0.032). The median AgI score was found to be higher for TH group in comparison to TN group (median 8 vs. 6) (Fig. 2). A significantly increased risk for tumor aggression was observed among the patients of TH group i.e., 3.33 times greater odds for aggressive and 11.58 times greater odds for highly aggressive tumor (*P* = 0.000) (Table 2). Multivariate analysis also showed the independent association of TH group with AgI having 2.469 times greater odds for AgI score 5–8 (95%CI: 0.514–11.857) and 6.919 times greater odds for AgI score > 8 (95%CI: 1.249–38.332, *P* = 0.021) (Table 3).

**Table 2:** Comparison of HCV Treatment Naïve and HCV-Treated HCC patients

Parameter		All HCV-HCC (***n*** = 171)	TN (***n*** = 120)	TH (***n*** = 51)	Crude Odds	95% CI	*P*-value^1^
Age (yr)	> 55	98 (57.3)	77 (64.2)	21 (41.2)	Reference	–	0.005
	≤ 55	73 (42.7)	43 (35.8)	30 (58.8)	2.56	1.30 to 5.00	
Sex	Female	63 (36.8)	41 (34.2)	22 (43.1)	Reference	–	0.266
	Male	108 (63.2)	79 (65.8)	29 (56.9)	0.68	0.35 to 1.34	
Tobacco	No	73 (42.7)	47 (39.2)	26 (51.0)	Reference	–	0.153
	Yes	98 (57.3)	73 (60.8)	25 (49.0)	0.62	0.32 to 1.19	
DM	No	118 (69.6)	87 (73.1)	31 (60.8)	Reference	–	0.110
	Yes	52 (30.4)	32 (26.9)	20 (39.2)	1.75	0.87 to 3.51	
TBIL (mg/dL)	≤ 1.5	76 (44.4)	51 (42.5)	25 (49.0)	Reference	–	0.432
	> 1.5	95 (55.6)	69 (57.5)	26 (51.0)	0.77	0.39 to 1.48	
Albumin (g/dL)	> 3.5	22 (12.9)	15 (12.5)	7 (13.7)	Reference	–	0.369
	2.8–3.5	94 (55.0)	70 (40.9)	24 (14.0)	0.74	0.26 to 2.02	
	< 2.8	55 (32.2)	35 (20.5)	20 (11.7)	1.22	0.43 to 3.51	
ALT (IU/L)	≤ 56	94 (55)	64 (53.3)	30 (58.8)	Reference	–	0.509
	> 56	77 (45)	56 (46.7)	21 (41.2)	0.80	0.41 to 1.55	
ALKP (IU/L)	≤ 125	26 (15.2)	16 (13.3)	10 (19.6)	Reference	–	0.296
	> 125	145 (84.8)	104 (86.7)	41 (80.4)	0.63	0.26 to 1.50	
Plt (× 10^9^/L)	≥ 150	57 (33.3)	48 (40.0)	9 (17.6)	Reference	–	0.005
	< 150	114 (66.7)	72 (60.0)	42 (82.4)	3.11	1.38 to 6.97	
NLR	≤ 2.5	57 (33.3)	46 (38.3)	11 (21.6)	Reference	–	0.033
	> 2.5	114 (66.7)	74 (61.7)	40 (78.4)	2.26	1.05 to 4.84	
CTP Class	Class A	33 (19.3)	25 (20.8)	8 (15.7)	Reference	–	0.257
	Class B	89 (52.0)	65 (54.2)	24 (47.1)	1.15	0.46 to 2.91	
	Class C	49 (28.7)	30 (25.0)	19 (37.3)	1.98	0.74 to 5.28	
Liver Size	Normal	51 (29.8)	37 (30.8)	14 (27.5)	Reference	–	0.386
	Enlarge	56 (32.7)	42 (35.0)	14 (27.5)	0.88	0.37 to 2.09	
	Decrease	64 (37.4)	41 (34.2)	23 (45.1)	1.48	0.67 to 3.29	
BCLC Stage ^2^	Stage A	27 (15.8)	20 (16.7)	7 (13.7)	Reference	–	0.032
	Stage B	17 (9.9)	11 (9.2)	6 (11.8)	1.56	0.418 to 5.80	
	Stage C	65 (38.0)	53 (44.2)	12 (23.5)	0.65	0.22 to 1.88	
	Stage D	62 (36.3)	36 (30.0)	26 (51.0)	2.06	0.76 to 5.59	
MTD (cm)	< 4.45	81 (47.4)	55 (45.8)	26 (51.0)	Reference	–	0.816
	4.45–9.6	73 (42.7)	53 (44.2)	20 (39.2)	0.80	0.40 to 1.60	
	> 9.6	17 (9.9)	5 (9.8)	12 (10.0)	0.88	0.28 to 2.76	
AFP (ng/dL)	< 100	74 (43.3)	55 (45.8)	19 (37.3)	Reference	–	0.049
	100–1000	63 (36.8)	47 (39.2)	16 (31.4)	0.985	0.46 to 2.13	
	> 1000	34 (19.9)	18 (15.0)	16 (31.4)	2.573	1.10 to 6.03	
PVT	No	120 (70.2)	96 (80.0)	24 (47.1)	Reference	–	0.000
	Yes	51 (29.8)	24 (20.0)	27 (52.9)	4.500	2.22 to 9.14	
Nodules	≤ 3	87 (50.9)	70 (58.3)	17 (33.3)	Reference	–	0.003
	> 3	84 (49.1)	50 (41.7)	31 (66.7)	2.800	1.41to 5.56	
AgI	Score = 4	22 (12.9)	20 (16.7)	2 (3.9)	Reference	–	0.000
	Score 5–8	108 (63.2)	81 (67.5)	27 (52.9)	3.33	0.73 to 15.20	
	Score > 8	41 (24.0)	19 (15.8)	22 (43.1)	11.58	2.39 to 56.09	

^1^*P*-value < 0.05 was considered as significant calculated by χ^2^–test

^2^There was not a single patient of stage 0 in our selected patients

*HCV* Hepatitis-C Virus; *HCC* Hepatocellular carcinoma; *TN* HCV Treatment Naïve; *TH* Treated for HCV infection using interferon/DAA based regimens; *DM* Diabetes Mellitus; *TBIL* Total bilirubin; *ALT* Alanine aminotransferase; *ALKP* Alkaline phosphatase; *Plt* Platelet count; *NLR* Neutrophil to lymphocyte ratio; *CTP class Child-*Turcotte*-Pugh class; BCLC* Barcelona-Clinic *Liver Cancer staging; MTD* Maximum tumor diameter; *AFP* Alpha-fetoprotein; *PVT* Portal vein thrombosis; *AgI* Aggressiveness Index (sum of score) = MTD (in tertiles): MTD < 4.5; 4.5 = MTD = 9.6; MTD > 9.6; scores 1, 2, 3 respectively; AFP (cut-off): AFP < 100; 100 = AFP = 1000; AFP > 1000; scores 1, 2, 3 respectively; PVT) (no/yes): PVT (no); PVT (yes); scores 1, 3 respectively; nodules (number): Nodules = 3; nodules > 3; scores 1, 3 respectively

**Table 3:** Multiple logistic regression analysis for HCV treatment effect on aggressiveness index score

Parameter^**1**^		Adj. Odds	S.E OR	95% CI	***P***-value^2^
Sex	Female	Reference	–	–	0.400
	Male	0.707	0.412	0.315 to 1.585	
Age (yr)	> 55	Reference	–	–	0.055
	≤ 55	2.064	0.397	0.983 to 4.336	
DM	No	Reference	–	–	0.219
	Yes	1.650	0.407	0.743 to 3.663	
Tobacco	No	Reference	–	–	0.194
	Yes	0.596	0.399	0.272 to 1.303	–
Plt (× 10^9^/L)	≤ 150	Reference	–	–	0.109
	< 150	2.023	0.451	0.836 to 4.894	–
NLR	≤ 2.5	Reference	–	–	0.429
	> 2.5	1.408	0.435	0.600 to 3.304	–
AgI	Score = 4	Reference	–	–	0.021
	Score 5–8	2.469	0.801	0.514 to 11.857	–
	Score > 8	6.919	0.873	1.249 to 38.332	–

^1^Overall Model was applied while considering HCV treatment naïve patients as reference ^2^ P-value < 0.05 was considered as significant

*HCV* Hepatitis-C Virus; *HCC* Hepatocellular carcinoma; *DM* Diabetes Mellitus; *Plt* Platelet count; *NLR* Neutrophil to lymphocyte ratio; *AgI* Aggressiveness Index (sum of score) = Maximum Tumor Diameter (MTD) (in tertiles): MTD < 4.5; 4.5 = MTD = 9.6; MTD > 9.6; scores 1, 2, 3 respectively; Alpha-fetoprotein (AFP) (cut-off): AFP < 100; 100 = AFP = 1000; AFP > 1000; scores 1, 2, 3 respectively; Portal Vein Thrombosis (PVT) (no/yes): PVT (no); PVT (yes); scores 1, 3 respectively; nodules (number): Nodules = 3; nodules > 3; scores 1, 3 respectively

### Impact of HCV treatment and tumor aggression on clinical parameters

Mean total bilirubin (mean ± S.D. = 3.61 ± 3.29, *P* = 0.001), platelet count (mean ± S.D. = 116.02 ± 73.13, *P* = 0.000) and NLR (mean ± S.D. = 4.61 ± 4.41,*P* = 0.012) were significantly higher in patients with highly aggressive tumor pattern (Table 4). Hypoalbuminemia was prominent in all the three categories, particularly in the highly aggressive group (mean ± S.D. = 2.83 ± 0.54, *P* = 0.477) (Table 4). The significantly associated parameters in Table 4 was considered to analyze the distribution in two groups i.e., in TH and TN groups and was assembled in Table 5. Patients with raised NLR were found to be in greater proportion in TH group as compared to TN group (54.1 vs. 45.9%). Similarly among the patients having highly aggressive tumor pattern, hypoalbuminemia was more prominent in TH group as compared to TN group (55.6 vs. 44.4%) (Table 5).

**Table 4:** Comparison of liver function parameters and lab values on basis of aggression index categories

Parameter	Non Aggressive (***n*** = 22)	Aggressive (***n*** = 108)	Highly Aggressive (***n*** = 41)	***P***-value^**1**^
	Mean ± S.D	Mean ± S.D	Mean ± S.D	
Age (yr)	58.09 ± 7.87	58.83 ± 8.36	54.59 ± 5.93	0.012^a,b^
TBIL (mg/dL)	1.73 ± 1.31	2.00 ± 1.67	3.61 ± 3.29	0.001^a,b^
Albumin (g/dL)	3.00 ± 0.53	2.95 ± 0.69	2.83 ± 0.54	0.477
CTP Score	7.50 ± 1.47	8.17 ± 1.96	9.07 ± 1.75	0.002^a,b^
ALT (IU/L)	61.55 ± 39.54	66.25 ± 41.73	77.59 ± 69.02	0.735
ALKP (IU/L)	249.50 ± 103.58	293.14 ± 179.52	333.66 ± 234.26	0.490
Plt (× 10^9^/L)	204.68 ± 93.06	148.85 ± 69.88	116.02 ± 73.13	0.000^a,b^
Neutrophils (%age)	61.00 ± 14.54	65.31 ± 10.26	70.54 ± 9.46	0.004^a,b^
Lymphocytes (%age)	28.91 ± 11.08	23.47 ± 9.52	20.66 ± 8.43	0.017^c^
NLR	2.64 ± 1.26	3.58 ± 2.29	4.61 ± 4.41	0.012^a,b^

^1^*P*-value < 0.05 was considered as significant calculated by Kruskal Wallis test

^a^ value is significant between Non aggressive and highly aggressive

^b^ Value is significant between Aggressive and highly aggressive

^c^ value is significant between Non aggressive vs. aggressive group and Non aggressive vs. highly aggressive group

*HCV* Hepatitis-C Virus; *HCC* Hepatocellular carcinoma; *TBIL* Total bilirubin; *CTP class Child-*Turcotte*-Pugh class;ALT* Alanine aminotransferase; *ALKP* Alkaline phosphatase; *Plt* Platelet count; *NLR* Neutrophil to lymphocyte ratio; No Aggression = AgI score = 4; Aggressive = AgI score 5–8; Highly Aggressive = AgI score > 8; AgI: Aggressiveness Index (sum of score) = MTD (in tertiles): MTD < 4.5; 4.5 = MTD = 9.6; MTD > 9.6; scores 1, 2, 3 respectively; AFP (cut-off): AFP < 100; 100 = AFP = 1000; AFP > 1000; scores 1, 2, 3 respectively; PVT) (no/yes): PVT (no); PVT (yes); scores 1, 3 respectively; nodules (number): Nodules = 3; nodules > 3; scores 1, 3 respectively

**Table 5:** Frequency distribution of age and different liver function parameters for aggressive tumor pattern in treated and non-treated groups

	TN ***n*** (%age)	TH ***n*** (%age)
**Age (Years)**		
**≤ 55**		
Non Aggressive	6 (100)	0 (0)
Aggressive	26 (63.4)	15 (36.6)
Highly Aggressive	11 (42.3)	15 (57.7)
**> 55**		
Non Aggressive	13 (81.25)	3 (18.75)
Aggressive	54 (80.6)	13 (19.4)
Highly Aggressive	10 (66.7)	5 (33.3)
**TBIL (mg/dL)**		
**≤ 1.5**		
Non Aggressive	11 (91.7)	1 (8.3)
Aggressive	37 (68.5)	17 (31.5)
Highly Aggressive	3 (30)	7 (70)
**> 1.5**		
Non Aggressive	9 (90.0)	1 (10.0)
Aggressive	44 (81.5)	10 (18.5)
Highly Aggressive	16 (51.6)	15 (48.4)
**CTP Class**		
**A**		
Non Aggressive	6 (100)	0 (0)
Aggressive	18 (75)	6 (25)
Highly Aggressive	1 (33.3)	2 (66.7)
**B**		
Non Aggressive	12 (85.7)	2 (14.3)
Aggressive	44 (75.9)	14 (24.1)
Highly Aggressive	9 (52.9)	8 (47.1)
**C**		
Non Aggressive	2 (100)	0 (0)
Aggressive	19 (73.1)	7 (26.9)
Highly Aggressive	9 (42.9)	12 (57.1)
**NLR**		
**≤ 2.5**		
Non Aggressive	9 (100)	0 (0)
Aggressive	35 (79.5)	9 (20.5)
Highly Aggressive	2 (50)	2 (50)
**> 2.5**		
Non Aggressive	11 (84.6)	2 (15.4)
Aggressive	46 (71.9)	18 (28.1)
Highly Aggressive	17 (45.9)	20 (54.1)
**Plt (× 10**^**9**^**/L)**		
**≥ 150**		
Non Aggressive	13 (92.9)	1 (7.1)
Aggressive	31 (83.8)	6 (16.2)
Highly Aggressive	4 (66.7)	2 (33.3)
**< 150**		
Non Aggressive	7 (87.5)	1 (12.5)
Aggressive	50 (70.4)	21 (29.6)
Highly Aggressive	15 (42.9)	20 (57.1)
**Albumin (g/dL)**		
**> 3.5**		
Non Aggressive	2 (100)	0 (0)
Aggressive	12 (70.6)	5 (29.4)
Highly Aggressive	1 (33.3)	2 (66.7)
**2.8–3.5**		
Non Aggressive	15 (88.2)	2 (11.8)
Aggressive	45 (78.9)	12 (21.1)
Highly Aggressive	10 (50)	10 (50)
**< 2.8**		
Non Aggressive	3 (100)	0 (0)
Aggressive	24 (70.6)	10 (29.4)
Highly Aggressive	8 (44.4)	10 (55.6)

*TN* HCV Treatment Naïve (*n* = 120); *TH* Treated for HCV infection using interferon/DAA based regimens (*n* = 51; Non Aggressive = AgI score = 4; Aggressive = AgI score 5–8; Highly Aggressive = AgI score > 8; *TBIL* Total bilirubin; *CTP class Child-*Turcotte*-Pugh class*; *Plt* Platelet count; *NLR* Neutrophil to lymphocyte ratio;

HCV Treatment Naïve Treated for HCV infection using interferon/DAA based regimens

### Impact of HCV treatment and tumor aggression on survival age of HCC patients

**Fig. 3:**
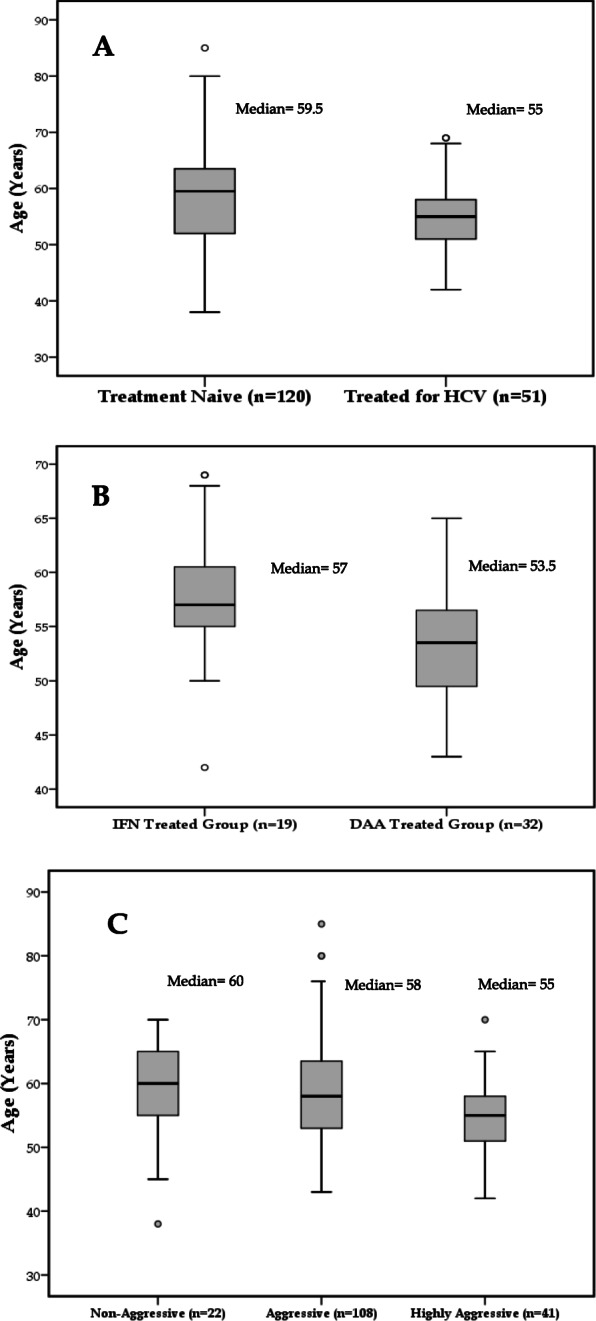
Age distribution with reference to Aggression Index (AgI) among HCV-related HCC patients. **a**: Distribution of age in treatment naïve and HCV treated patients; **b:** Distribution of age among treated group who receive interferon and direct acting anti-viral (DAAs) agents; **c:** Distribution of age among aggression index categories; Not Aggressive = AgI score 4; Aggressive = AgI score 5–8; Highly Aggressive = AgI score > 8

Overall mean age in HCV-related HCC patients was 57.72 ± 7.95 (Table 1) and median age of TN group was higher as compared to TH group (59.5 vs. 55 years) (Fig. 3a). Patients in DAA treated group were found to be younger as compared to IFN treated group (53.5 vs. 57 years) at the time of HCC diagnosis (Fig. 3b). The relative proportion of younger subjects (≤ 55 year) was also higher in TH group as compared to TN group (58.8 vs. 35.8%) (Table 2). Similarly, the patients who presented with highly aggressive tumor pattern were significantly younger i.e., (mean ± S.D. = 54.59 ± 5.93, *P* = 0.012) (Table 4; Fig. 3c). All patients of ≤ 55 years in TH group were presented with tumor aggression (AgI score > 4) with a greater proportion in highly aggressive pattern as compared to TN group (57.7 vs. 42.3%) (Table 5).Furthermore, treated male patients were significantly in greater proportion in younger age group as compared to older ones (62.1 vs. 37.9%, *P* = 0.049) (Table 6).

**Table 6:** Association of gender with age in treated and non-treated groups

	Age (Years)	TN ***n*** (%age)	TH ***n*** (%age)	***P***-value^**1**^
**Male**	≤ 55	31 (39.2)	18 (62.1)	0.049
	> 55	48 (60.8)	11(37.9)	
**Female**	≤ 55	12 (29.3)	12 (54.5)	0.061
	> 55	29 (70.7)	10 (45.5)	

^1^*P*-value < 0.05 was considered as significant calculated by *x*^2^ -test

*TN* HCV Treatment Naïve (*n* = 120); *TH* Treated for HCV infection using interferon/DAA based regimens (*n* = 51);

## Discussion

Out of various etiological factors, HCV-infection is gaining importance as a major cause of HCC [41]. After introduction of anti-HCV therapy, enormous disease burden is reduced, especially in terms of liver failure and HCC incidence [30, 31]. Complete viral eradication and SVR had been reported to significantly reduce HCC cases, however risk persisted even after 10-years of viral eradication [42]. HCC patients early diagnosed showed a better overall survival compared to late stage or aggressive tumor presentation [3, 43]. Guidelines provided by EASL and AASLD recommend screening program for early diagnosis of high-risk patients. Great success has already been shown in screening program of Japan [44].

The possible association of anti-HCV therapy, especially DAA, with HCC is actively debated. Several studies have highlighted the correlation of anti-HCV treatment with HCC onset and recurrence, however, the tumor pattern after anti-viral therapy has not been analyzed. Then, we focused our attention on characterizing this aspect. Tumor pattern was analyzed through aggressiveness index scoring system which has been shown to have clinical utility in predicting the patient’s survival and extent of disease [8, 9, 45]. In this study, a tumor pattern more aggressive than usual was observed among HCV treated patients (*P*-value = 0.021) (Table 3). Moreover, for TH group the odds for aggressive and highly aggressive tumors were 2.469 and 6.919 times, respectively, greater in comparison to TN group (Table 3). A study carried on a larger cohort (*n* = 362) showed that all four AgI parameters were independent predictors of patients survival [46]. Similarly, PVT in HCC patients has been shown to be associated with marked decrease in patients’ survival [46] and we observed a significantly greater proportion of PVT in TH group as compared to TN group (52.9 *vs.* 20%, *P* = 0.000) (Table 2). Recently, Romano et al. [47] also demonstrated an aggressive tumor after HCV therapy in the form of higher number of nodules and extra-hepatic metastases, suggesting that the tumor growth in such patients is faster than usual. A controversial finding was also reported, where Reig et al. [48] showed that recurrence of HCC among patients who took DAA therapy was more aggressive in comparison with initial tumor patterns while Conti et al. [36] reported no difference in recurrent tumor pattern. In another study conducted by Abdelaziz et al., [49] patterns of tumor occurrence or recurrence in patients who received DAA were characterized by higher α-fetoprotein levels along with more infiltrative pattern indicating the presence of significant lymphadenopathy and malignant PVT among treated patients.

The enrollment of 2.4 times greater HCC patients in TN group as compared to TH group during study period signifies the importance of early HCV screening and treatment because treatment naïve status is itself a greater risk for HCC [42]. In the instant study, majority of the enrolled patients were diagnosed symptomatically, while few were diagnosed during the screening (85.4 vs. 5.2%), with poor follow-up details for the TH group (Table 1). Shorter screening intervals in post HCV treated patients have been shown to reduce overall mortality in a dose-dependent manner [50]. In this study a strong association between BCLC stage and HCV treatment status (*P*-value = 0.032) (Table 2) was observed that indicates an urgent need for surveillance program at Pakistan for post-HCV treated patients for early detection of HCC which in turn decreases the mortality rate of HCV-related HCC. Moreover, the duration after anti-HCV therapy was found to be shorter among DAA treated patients as compared to IFN-treated patients (13.05 ± 7.35 vs.70.74 ± 15.03) (Table 1). This smaller duration with DAA treatment was consistent with findings of Ooka et al. [51] who reported that very early occurrence of HCC after DAA therapy is associated with prior appearance of imaging dysplastic nodules and hence critically confirming the existence of dysplastic nodule before DAA therapy would be useful to detect high-risk patients for very early HCC occurrence. Similarly, another study reported time association between HCC occurrence and start of interferon free anti-viral treatment with a median of 10.3 months [52].

In tumors that arise in the background of chronic inflammation such as HCV related HCC, the overall effect of immune system seems to stimulate tumor growth and progression [53]. Raised inflammatory markers have also been shown in association with HCC aggressiveness biology with an independent influence on prognosis of disease [54]. NLR reflects the systemic inflammation and is being shown in association with aggressive HCC phenotype [55]. In this study the greater odds (1.408) for raised NLR was observed for TH group (Table 3). NLR was also found to be significantly raised in patients presented with highly aggressive tumor pattern (*P*-value = 0.012) (Table 4). Hypoalbuminemia a prognostically important inflammatory index for many tumors, including HCC [54, 56], was observed among patients having highly aggressive tumor pattern (mean ± S.D. = 2.83 ± 0.054). The greater proportion of patients having highly aggressive tumor pattern with raised NLR and hypoalbuminemia in TH group as compared to TN group (54.1 vs. 45.9% and 55.6 *vs.* 44.4%, respectively) shows that highly aggressive HCC phenotype is associated with high levels of inflammatory markers (Table 5). Another study also concluded that HCC pattern is not independent of underlying liver microenvironment [45]. Inference of biological mechanisms associated with our findings is complex however, Conti et al. [36] discussed that rapid reduction of inflammation through DAA therapy rapidly reduces hepatic inflammation which decreases the activity of natural killer cells which are proposed to play a key role in immunosurveillance of neoplastic clone [57]. Therefore, it is suggested that the use of serum biomarkers for HCC detection and follow-up is becoming a vital necessity.

Age is being considered as a major determinant for HCC development after viral eradication [58, 59]. Generally HCC appears after twenty or more years of HCV infection [60]. This may also be considered as an indicator of duration of hepatitis, not only as marker of accumulated tissue and genetic injury but also the aging process. Among all HCV-related HCC patients, the average age was less 57.7 years (Table 1) which could also be attributed to small average age of Pakistani population i.e., 66.8 years as compared to other countries [61]. We observed a contradictory trend for age among TH group where proportion of patients in younger age group (≤ 55 years) was found to be greater as compared to TN group (58.8 vs. 35.8%)(Table 2, Fig. 3a).Non-aggressive HCC pattern was found to be prominent in TN group while all patients of TH group having younger age (≤ 55 years) were presented with aggressive tumor pattern (AgI > 4) with marginally greater proportion for highly aggressive pattern as compared to TN group (57.7 vs. 42.3%) (Table 5).The association of tumor aggression and DAA therapy with presentation at early age (supplementary Table 1, Fig. 3b) throws light on unidentified interaction between DAA and tumor patterns. This association could also be explained through patient related genetic factors in progression of tumor. Moreover, in TH group male patients have significantly greater proportion of younger age group as compared to older age group (62.1 vs. 37.9%, *P*-value 0.049) (Table 6) which could be explained through androgen associated up-regulation of hepatic inflammation and advanced fibrosis [62] with an exacerbation in TH patients having raised NLR. Scientists also explained the gender disparity in pathogenesis of CHC and HCC in terms of altered estrogen receptors [63] and increased testosterone levels [62] in male gender. Furthermore, it has been demonstrated that in younger females (premenopausal state) the circulating estrogen is associated with less severity and slow progression of CHC infection into fibrosis and HCC as compared to postmenopausal females who lack circulating estrogen [64]. However, a large cohort is required to draw a specific conclusion for association of age, gender and HCV treatment.

The enrolment of one third proportion of HCV treated patients during the instant study period supports the success of anti-HCV therapy. However, highly aggressive tumor, elevated NLR and hypoalbuminemia in majority of TH patients is a striking observation which needs to be explained in terms of interaction between liver damage and HCC biology, inflammatory cascade [65], HCV infection relapse with possibility of emergence of more virulent strain of HCV [66, 67] to avoid any undue negative impression about the gold standard anti-HCV drugs that have saved millions of the people. Moreover, observations of this study do not allow us to surmise an interaction between HCV treatment and tumor related factors that only the HCC, if develop after HCV treatment, is associated with aggressive tumor pattern. In a recent study, scientists demonstrated that DAA mediated vascular endothelial growth factor (an angiogenesis inducer) which acts as a triggering factor for neo-angiogenetic pathway was elevated among patients who developed de novo HCC after DAA therapy [68]. Similarly, in another study scientists suggested that few clones are primed to grow and become cancer in small but clinically relevant proportion of patients, hence achievement of SVR should not be regarded as a role towards the HCC development instead the key is to find up to what extent anti-HCV therapy is involve [52]. Additionally, some studies have reported that DAA have little role in cirrhotic and HCC patients with markedly decreased survival time in HCC cirrhotic patients as compared to non-treated HCV-HCC patients [36, 48, 69]**.** The observations reported in these studies are not commenting on the efficacy of DAAs but highlight the need to cautiously prescribe DAAs in HCC patients. Moreover, clinicians and patients should be informed that anti-HCV therapy does not substitute the necessity of surveillance as the risk to develop HCC remains even after achieving SVR.

This study also has substantial limitations, including the uncertainty for the duration between onset of HCV infection and HCC development in selected patients. Secondly, majority of patients were diagnosed for HCC when their disease was symptomatic. Additionally, at the time of start of anti-HCV therapy, the damaged cause by CHC, state of immune response and stage of HCC was not taken into consideration. Hence, the data from larger cohorts which addresses these limitations should be sought to confirm findings of this study.

## Conclusion and recommendations

Despite the deployment of newer DAAs, HCV-related HCC will remain a major health issue in coming decades. Due to urgent unmet needs for early HCC detection and intervention, post-treatment HCV related HCC is found to be an emerging problem. We observed that aggressive and highly aggressive tumor were more prominent in TH patients, which need to be understood through a prospective study on large cohort and demands preemptive actions for screening of HCC in HCV treated patients through public or industry supported pharmacovigilance programs. Moreover, it is recommended that anti-HCV therapy may be deferred in HCC patients until a clear risk to benefit ratio is defined through further studies.

## Supplementary information

**Additional file 1: Table S1.** Comparison of HCV-related HCC Patients who received IFN and DAA therapy. **Table S2.** HCV anti-body test details ^1^.

## Data Availability

Most of the data generated or analyzed during this study are included within the article and its additional files. The datasets used and/or analyzed during the currentstudy are available with the corresponding author and may be obtained on proper/reasonable request.

## Abbreviations

AASLD: American Association for the Study of Liver Diseases
AFP: Alpha-fetoprotein
AgI: Aggression Index
ALKP: Alkaline phosphatase
ALT: Alanine aminotransferase
AST: Aspartate transaminase
BCLC: Barcelona Clinic Liver Cancer
CHC: Chronic hepatitis C
cOR: Crude odds
CTP class: *Child-*Turcotte*-Pugh class*
DAAs: Direct acting antiviral agents
DM: Diabetes Mellitus
EASL: European Association for the Study of the Liver
HBV: Hepatitis B virus
HCC: Hepatocellular carcinoma
HCV: Hepatitis C virus
IFN: Interferon
MTD: Maximum tumor diameter
NLR: Neutrophil to lymphocyte ratio
Plt: Platelet count
PVT: Portal vein thrombosis
SVR: Sustained viral response
TBIL: Total bilirubin
TH: Treated for HCV infection
TN: HCV treatment naïve
US: Ultrasound
