# Supplementary material for: Tumor aggression among hepatitis-C related hepatocellular carcinoma patients: an observational study regarding the impact of anti-HCV therapy
Source: Infect Agent Cancer. 2020 May 27;15:35. doi: 10.1186/s13027-020-00300-z (PMC7251734; doi:10.1186/s13027-020-00300-z)
Supplement: Supplementary file 1 — Additional file 1: Table S1. Comparison of HCV-related HCC Patients who received IFN and DAA therapy. Table S2. HCV anti-body test details 1. [file 13027_2020_300_MOESM1_ESM.docx]

**Supplementary Table 1 Comparison of HCV-related HCC Patients who received IFN and DAA therapy**

| **Parameter** |  | **TH**  **(n=51)** | **IFN Treated (n = 19)** | **DAA Treated (n = 32)** | **Crude Odds** | **95% CI** | **P-value^1^** |
| --- | --- | --- | --- | --- | --- | --- | --- |
| Age (yr) | > 55 | 21 (41.2) | 12 (63.16) | 9 (28.13) | Reference | - | 0.014 |
|  | ≤ 55 | 30 (58.8) | 7 (36.84) | 23 (71.88) | 4.38 | 1.30 to 14.68 |  |
| Sex | Female | 22 (43.1) | 8 (42.11) | 14 (43.8) | Reference | - | 0.909 |
|  | Male | 29 (56.9) | 11 (57.89) | 18 (56.2) | 0.94 | 0.30 to 2.95 |  |
| Tobacco | No | 26 (51.0) | 9 (47.37) | 17 (56.1) | Reference | - | 0.691 |
|  | Yes | 25 (49.0) | 10 (52.63) | 15 (46.9) | 0.80 | 0.25 to 2.48 |  |
| DM | No | 31 (60.8) | 12 (63.2) | 19 (59.4) | Reference | - | 0.789 |
|  | Yes | 20 (39.2) | 7 (36.8) | 13 (40.6) | 1.17 | 0.36 to 3.78 |  |
| TBIL (mg/dL) | ≤ 1.5 | 25 (49.0) | 8 (42.1) | 17 (53.1) | Reference | - | 0.447 |
|  | > 1.5 | 26 (51.0) | 11 (57.9) | 15 (46.9) | 0.64 | 0.20 to 2.02 |  |
| Albumin (g/dL) | > 3.5 | 7 (13.7) | 3 (15.8) | 4 (12.5) | Reference | - | 0.528 |
|  | 2.8-3.5 | 24 (47.1) | 7 (36.8) | 17 (53.1) | 1.82 | 0.16 to 5.21 |  |
|  | < 2.8 | 20 (39.2) | 9 (47.4) | 11 (34.4) | 0.92 | 0.32 to 10.34 |  |
| ALT (IU/L) | ≤ 56 | 30 (58.8) | 7 (36.8) | 23 (71.9) | Reference | - | 0.014 |
|  | > 56 | 21 (41.2) | 12 (63.2) | 9 (28.1) | 0.23 | 0.07 to 0.77 |  |
| ALKP (IU/L) | ≤ 125 | 10 (19.6) | 4 (21.1) | 6 (18.8) | Reference | - | 0.841 |
|  | >125 | 41 (80.4) | 15 (78.9) | 26 (81.2) | 1.16 | 0.28 to 4.76 |  |
| Plt (×10^9^/L) | > 150 | 9 (17.6) | 2 (10.5) | 7 (21.9) | Reference | - | 0.304 |
|  | ≤ 150 | 42 (82.4) | 17 (89.5) | 25 (88.1) | 0.42 | 0.08 to 2.27 |  |
| NLR | ≤ 2.5 | 11 (21.6) | 4 (21.1) | 7 (21.9) | Reference | - | 0.945 |
|  | > 2.5 | 40 (78.4) | 15 (78.9) | 25 (78.1) | 0.95 | 0.24 to 3.81 |  |
| CTP Class | Class A | 8 (15.7) | 2 (10.5) | 6 (18.8) | Reference | - | 0.703 |
|  | Class B | 24 (47.1) | 9 (47.4) | 15 (46.9) | 0.56 | 0.09 to 3.37 |  |
|  | Class C | 19 (37.3) | 8 (42.1) | 11 (34.4) | 0.46 | 0.07 to 2.89 |  |
| Liver Size | Normal | 14 (27.5) | 5 (26.3) | 9 (28.1) | Reference | - | 0.969 |
|  | Enlarge | 14 (27.5) | 5 (26.3) | 9 (28.1) | 1.00 | 0.21 to 4.69 |  |
|  | Decrease | 23 (45.1) | 9 (47.4) | 14 (43.8) | 0.86 | 0.212 to 3.43 |  |
| BCLC Stage ^2^ | Stage A | 7 (13.7) | 3 (15.8) | 4 (12.5) | Reference | - | 0.672 |
|  | Stage B | 6 (11.8) | 1 (5.3) | 5 (15.6) | 3.75 | 0.27 to 51.37 |  |
|  | Stage C | 12 (23.5) | 4 (21.1) | 8 (25.0) | 1.50 | 0.22 to 10.22 |  |
|  | Stage D | 26 (51.0) | 11 (57.9) | 15 (46.9) | 1.02 | 0.19 to 5.53 |  |
| MTD (cm) | < 4.45 | 26 (51.0) | 10 (52.6) | 16 (50.0) | Reference | - | 0.963 |
|  | 4.45-9.6 | 20 (39.2) | 7 (36.8) | 13 (40.6) | 1.16 | 0.35 to 3.90 |  |
|  | > 9.6 | 5 (9.8) | 2 (10.5) | 3 (9.4) | 0.94 | 0.13 to 6.63 |  |
| AFP (ng/dL) | ≤ 100 | 19 (37.3) | 7 (36.8) | 12 (37.5) | Reference | - | 0.343 |
|  | 100-1000 | 16 (31.4) | 4 (21.1) | 12 (37.5) | 1.75 | 0.40 to 7.58 |  |
|  | > 1000 | 16 (31.4) | 8 (42.1) | 8 (25.0) | 0.58 | 0.15 to 2.26 |  |
| PVT | No | 24 (47.1) | 9 (47.4) | 15 (46.9) | Reference | - | 0.973 |
|  | Yes | 27 (52.9) | 10 (52.6) | 17 (53.1) | 1.02 | 0.33 to 3.18 |  |
| Nodules | ≤ 3 | 17 (33.3) | 9 (47.4) | 8 (25.0) | Reference | - | 0.101 |
|  | > 3 | 34 (66.6) | 10 (42.5) | 24 (60.8) | 2.70 | 0.81 to 9.01 |  |
| AgI^3^ | Score = 4 | 2 (3.9) | 1 (5.3) | 1 (3.1) | - | - | 0.757 |
|  | Score 5-8 | 27 (52.9) | 11 (57.9) | 16 (50.0) | - | - |  |
|  | Score > 8 | 22 (43.1) | 7 (36.8) | 15 (46.9) | - | - |  |

^1^ ᵪ^2^ -test

^2^ There was not a single patient in stage 0 in our selected patients.

^3^ Crude odds was not calculated because of small number of patients in AgI group having score = 4

TH = Treated for HCV infection using interferon/DAA based regimens (n = 51); IFN: Interfero-α; DAA: Direct Acting Anti-viral agents (including sofosbuvir and daclatasvir); DM: Diabetes Mellitus; TBIL: Total bilirubin; ALT: Alanine aminotransferase; ALKP: Alkaline phosphatase; Plt: Platelet count; NLR: Neutrophil to lymphocyte ratio; CTP class: Child*-*Turcotte*-*Pugh class; BCLC: Barcelona Clinic Liver Cancer staging; MTD: Maximum tumor diameter; AFP: Alpha-fetoprotein; PVT: Portal vein thrombosis; AgI: Aggressiveness Index; Aggressiveness Index (sum of score) = MTD (in tertiles): MTD < 4.5; 4.5 = MTD = 9.6; MTD > 9.6; scores 1, 2, 3 respectively; AFP (cut-off): AFP < 100; 100 = AFP = 1000; AFP > 1000; scores 1, 2, 3 respectively; PVT) (no/yes): PVT (no); PVT (yes); scores 1, 3 respectively; nodules (number): Nodules ≤ 3; Nodules > 3; scores 1, 3 respectively.

**Supplementary Table 2 HCV anti-body test details ^1^**

| **Name of Kit** | **Sensitivity% (95% CI)** | **Specificity% (95% CI)** | **Manufacturer** |
| --- | --- | --- | --- |
| Imu Med (HCV Rapid Test Cassette) | 99.1 (97.8~99.8) | 99.6 (99.2~99.9) | Moon Enterprises |
| Advanced Quality (One Step HCV Test) | 97.1(89.8- 99.6) | 96.3 (92.5 - 98.5) | Bionike Inc. |
| RapiCard ™ InstaTest (One step HCV) | 100 | 99 | Cortez Diagnostics Inc. |

**1** The patient have been diagnosed in different hospitals/clinics at the time of diagnosis of the HCV. However the sensitivity and specificity of the HCV antibodies screening tests available in medical record of the patients is included here.
